# A176 SEROLOGICAL HEAVY METAL ASSESSMENT IN PERSONS WITH IBD

**DOI:** 10.1093/jcag/gwae059.176

**Published:** 2025-02-10

**Authors:** S Narvey, C L Dolovich, C N Bernstein

**Affiliations:** Medicine, University of Manitoba Max Rady College of Medicine, Winnipeg, MB, Canada; Internal Medicine, University of Manitoba Max Rady College of Medicine, Winnipeg, MB, Canada; University of Manitoba Max Rady College of Medicine, Winnipeg, MB, Canada

## Abstract

**Background:**

Inflammatory Bowel Disease (IBD), including Crohn’s disease (CD) and ulcerative colitis (UC), are chronic conditions of aberrant gut inflammation. The increasing prevalence of IBD in parallel with increasing human exposure to heavy metals (HMs) has raised concern regarding the contribution of HMs to gut pathology. Emerging evidence has implicated HMs as influential factors in IBD pathogenesis, however, our current understanding of how HMs impact gut barrier integrity, inflammation, and dysbiosis is limited in the context of IBD and deserving of further attention.

**Aims:**

The preliminary study aim was to establish a foundational understanding of serum levels of certain HMs in IBD. Additionally, we aimed to characterize a correlation between HM concentrations in serum and gut tissue.

**Methods:**

Using Inductively Coupled Plasma Mass Spectrometry (ICP-MS), serum concentrations of Aluminum (Al), Chromium (Cr), Mercury (Hg), and Titanium (Ti) were measured in individuals with CD, UC, multiple sclerosis (MS), and in healthy controls (HC), where MS was used as an immune-mediated inflammatory disease control. A bivariate analysis was used to evaluate associations between HM levels and specific disease groups. Logistic regression analysis was used to assess HM outcomes predicted by disease groups and healthy controls while controlling for demographic factors.

**Results:**

Relative to HCs, the proportion of higher serum levels of Al (79% vs. 38.6%, p<0.001), and Hg (63% vs 37.2%, p<0.001) were greater in CD. Whereas higher Ti levels were lower in CD (59%), UC (14.0%), and MS (6.1%) relative to HCs (84.1%); p-values <0.0002, <0.0001, and 0.001 respectively. In MS, the proportion of higher levels of Cr were greater compared to HCs (83.7% vs 38.0%, p<0.001) and Hg (57.1% vs 37.2%,p=0.025).When controlling for demographic factors, the odds of having higher serum Al (OR=6.41[CB1], 95%CI 3.04-13.5) and Hg (OR=2.27, 95%CI 1.13-4.57) were significantly greater in CD relative to HCs. In contrast, the odds of having elevated serum Ti were significantly lower in CD (OR=0.25, 95%CI 0.11-0.57) and UC (OR=0.02, 95%CI 0.01-0.08) relative to HCs. The odds of having increased Cr were 12 times greater for MS relative to HC [OR=12.33, 95%CI (3.83-39.7)]. In our investigation of gut biopsies, we did not find a correlation between HM levels in serum and intestinal tissue.

**Conclusions:**

Our investigation demonstrated significant differences in serum HM levels between CD, UC, MS, and HCs. These results support the need for further exploration into the specific involvement of HMs in IBD.

**Funding Agencies:**

None

